# Supplementary material for: Analysis of spliceosome-related coding and noncoding genes and pseudogenes reveals novel candidates
Source: medRxiv. 2026 Aug 10:2026.08.06.26358951. Preprint. [Version 1] doi: 10.64898/2026.08.06.26358951 (PMC13640761; doi:10.64898/2026.08.06.26358951)
Supplement: Supplementary file 1 [file NIHPP2026.08.06.26358951v1-supplement-2.pdf]

medRxiv preprint doi: https://doi.org/10.64898/2026.08.06.26358951; this version posted August 10, 2026. The copyright holder for this preprint (which was not certified by peer review) is the author/funder, who has granted medRxiv a license to display the preprint in perpetuity.
It is made available under a CC-BY 4.0 International license .

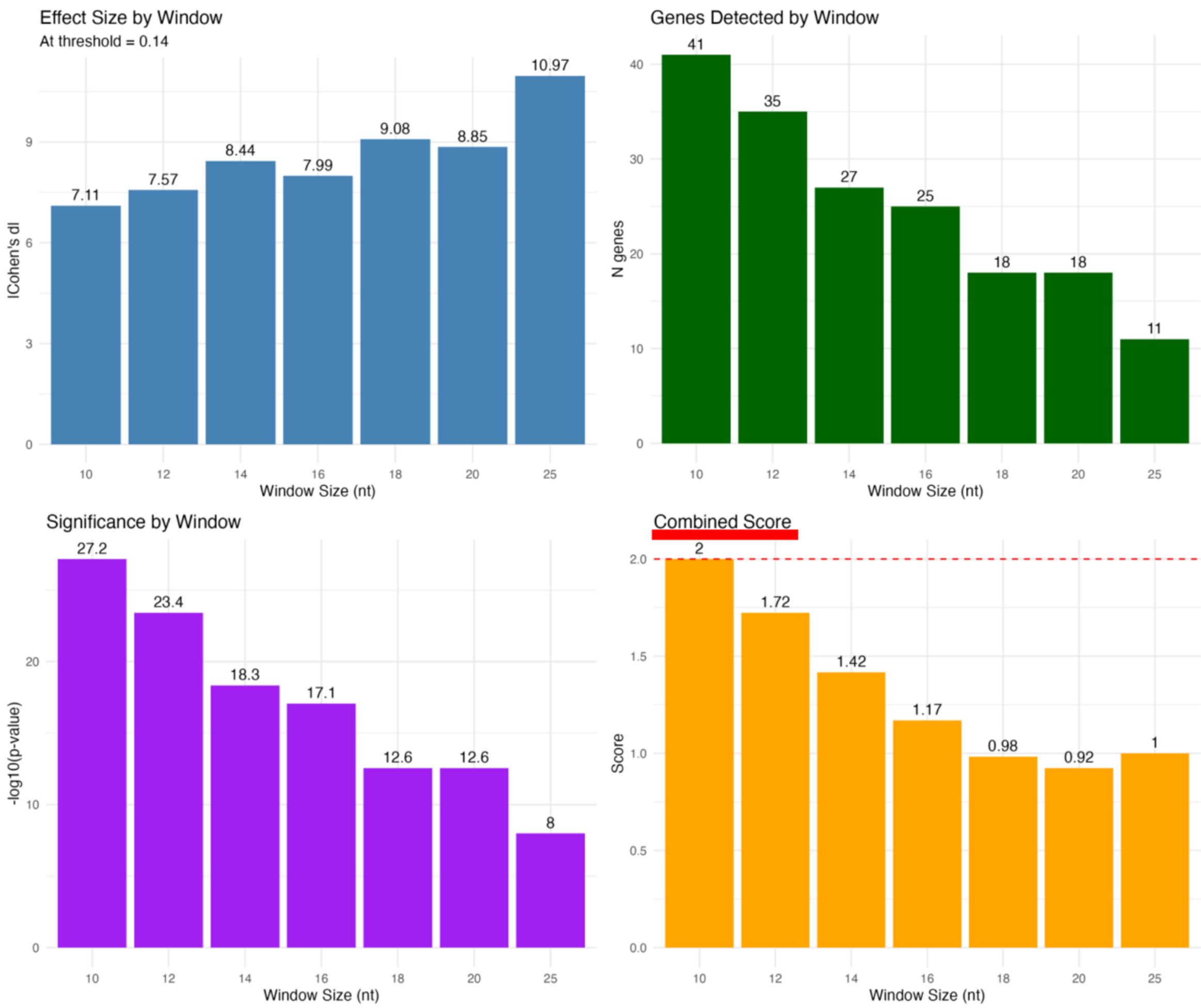

Supplementary Figure 1: Results of the statistical analysis at threshold 0.14. The number of genes detected (top right), the effect size (Cohen's d) (top left), the statistical significance (-log10 p-value) (bottom left), and the combined score (bottom right) by window size.

medRxiv preprint doi: https://doi.org/10.64898/2026.08.06.26358951; this version posted August 10, 2026. The copyright holder for this preprint (which was not certified by peer review) is the author/funder, who has granted medRxiv a license to display the preprint in perpetuity.
It is made available under a CC-BY 4.0 International license .

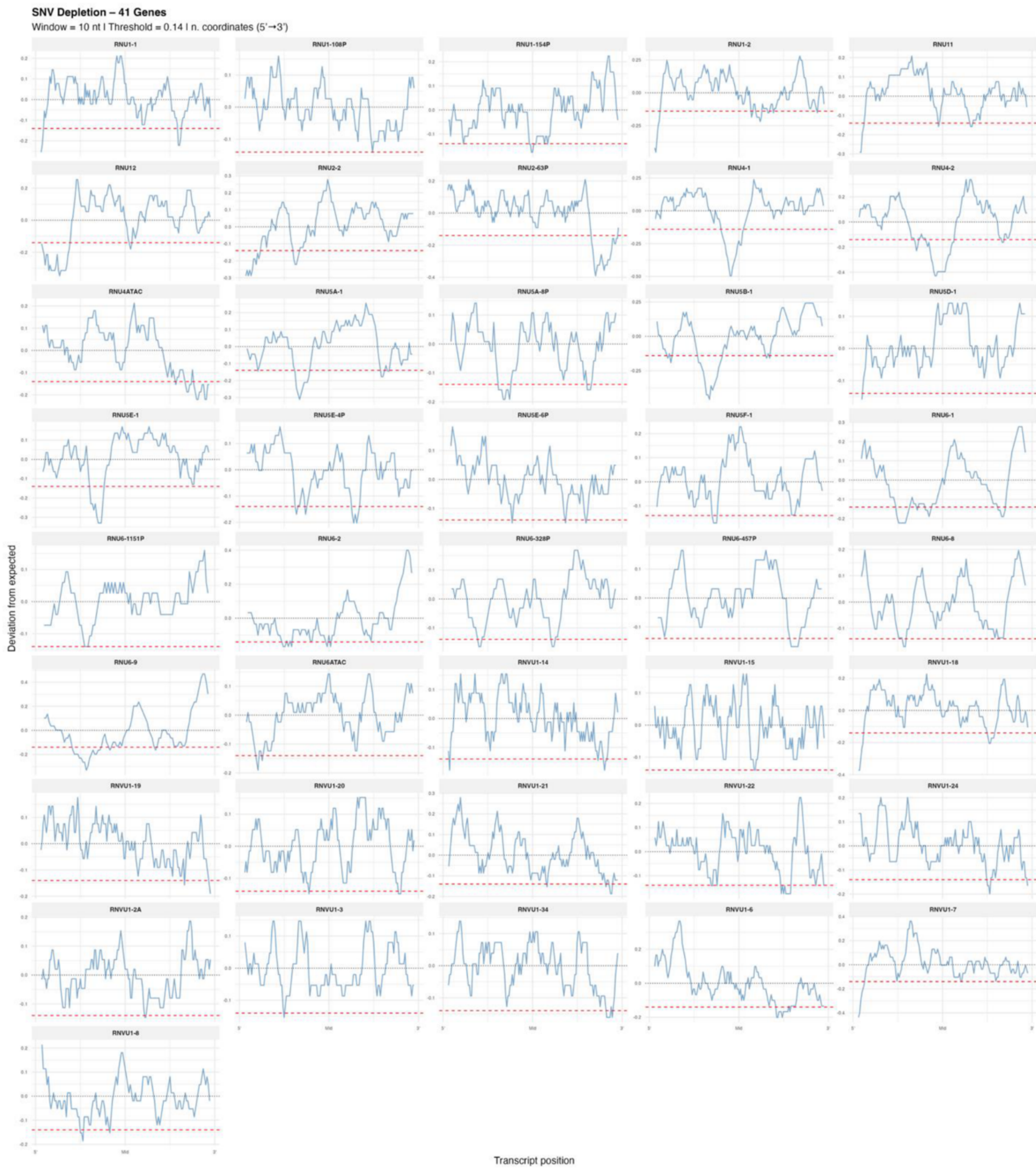

Supplementary Figure 2: Visualization of depleted regions for all prioritized genes. Plots show the distance to expected by normalized position. The dashed red line indicates the optimal threshold at 0.14.

medRxiv preprint doi: https://doi.org/10.64898/2026.08.06.26358951; this version posted August 10, 2026. The copyright holder for this preprint (which was not certified by peer review) is the author/funder, who has granted medRxiv a license to display the preprint in perpetuity.
It is made available under a CC-BY 4.0 International license .

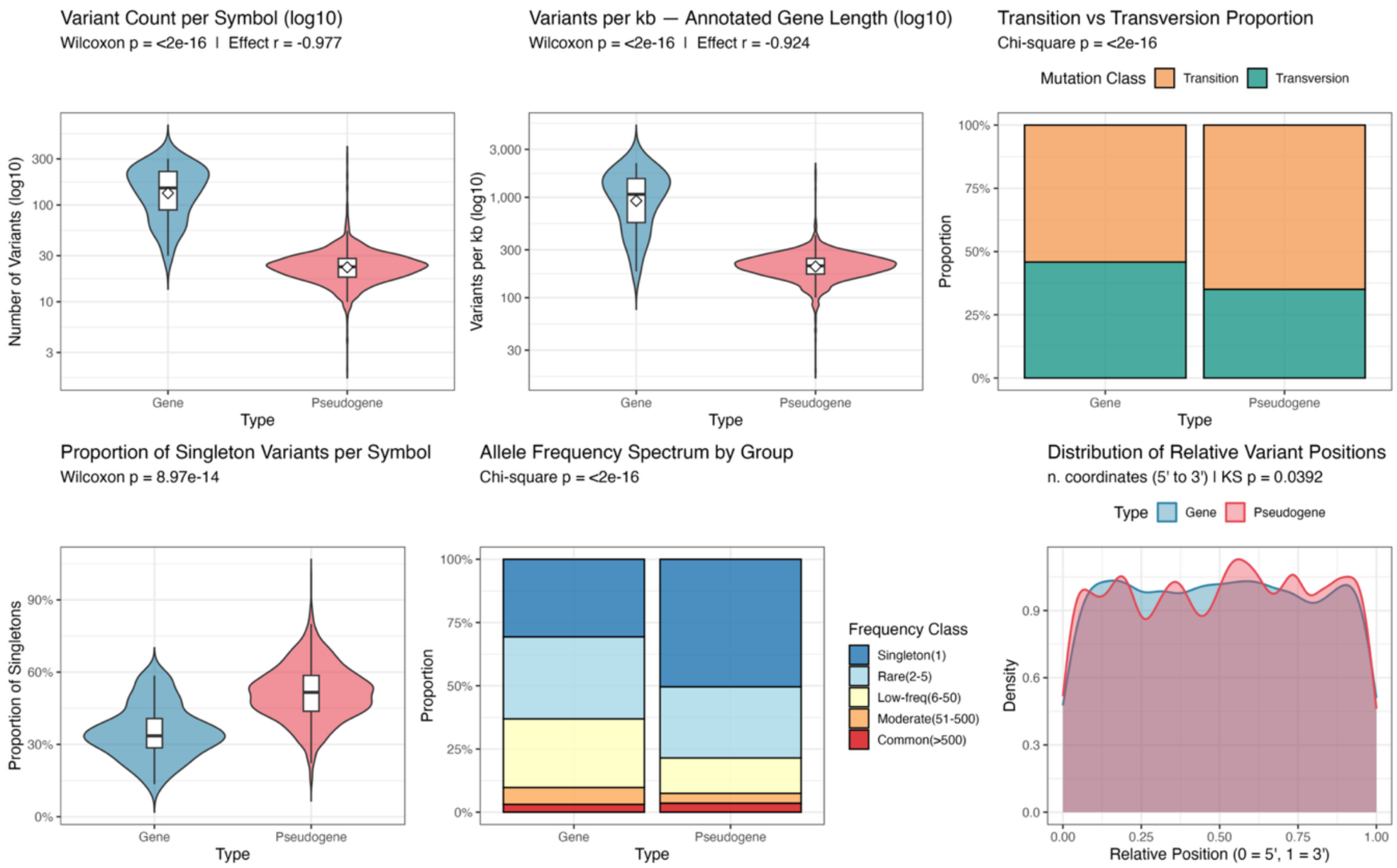

Supplementary Figure 3: Comparison between snRNA genes and pseudogenes. Plots show that snRNA genes have significantly higher number of variants than pseudogenes per gene (top left) and per kb (top middle), a significant difference in transition vs transversion proportion (top right), singleton proportion (bottom left), and allele frequency (bottom middle). Variant position distribution showed a significant difference in the distribution uniformity (bottom right).

medRxiv preprint doi: https://doi.org/10.64898/2026.08.06.26358951; this version posted August 10, 2026. The copyright holder for this preprint
(which was not certified by peer review) is the author/funder, who has granted medRxiv a license to display the preprint in perpetuity.
It is made available under a CC-BY 4.0 International license .

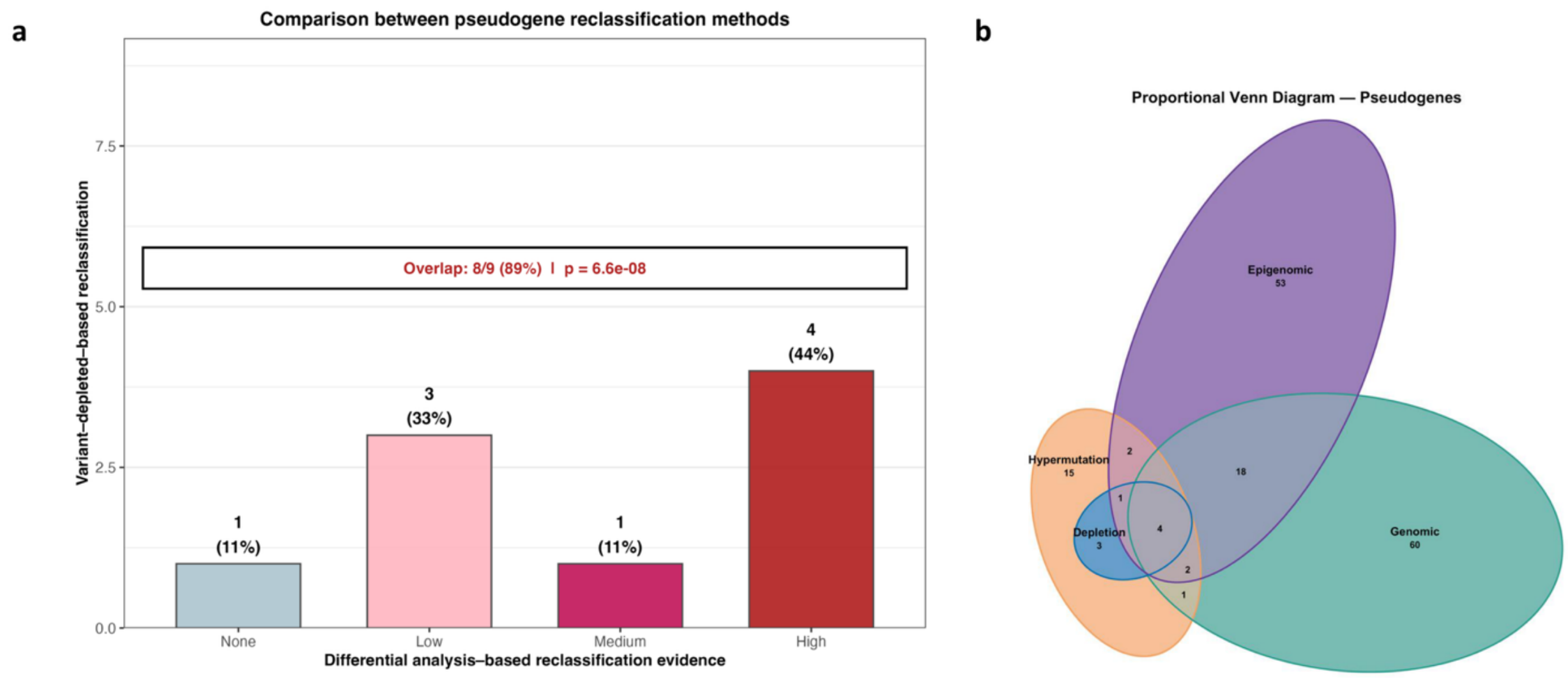

Supplementary Figure 4: a) **comparison between the variant-depletion approach and the differential approach.** b) **Proportional Venn diagram**. The numbers indicate the gene-like results by the different features of the differential analysis and the depletion analysis.

medRxiv preprint doi: https://doi.org/10.64898/2026.08.06.26358951; this version posted August 10, 2026. The copyright holder for this preprint (which was not certified by peer review) is the author/funder, who has granted medRxiv a license to display the preprint in perpetuity.
It is made available under a CC-BY 4.0 International license .

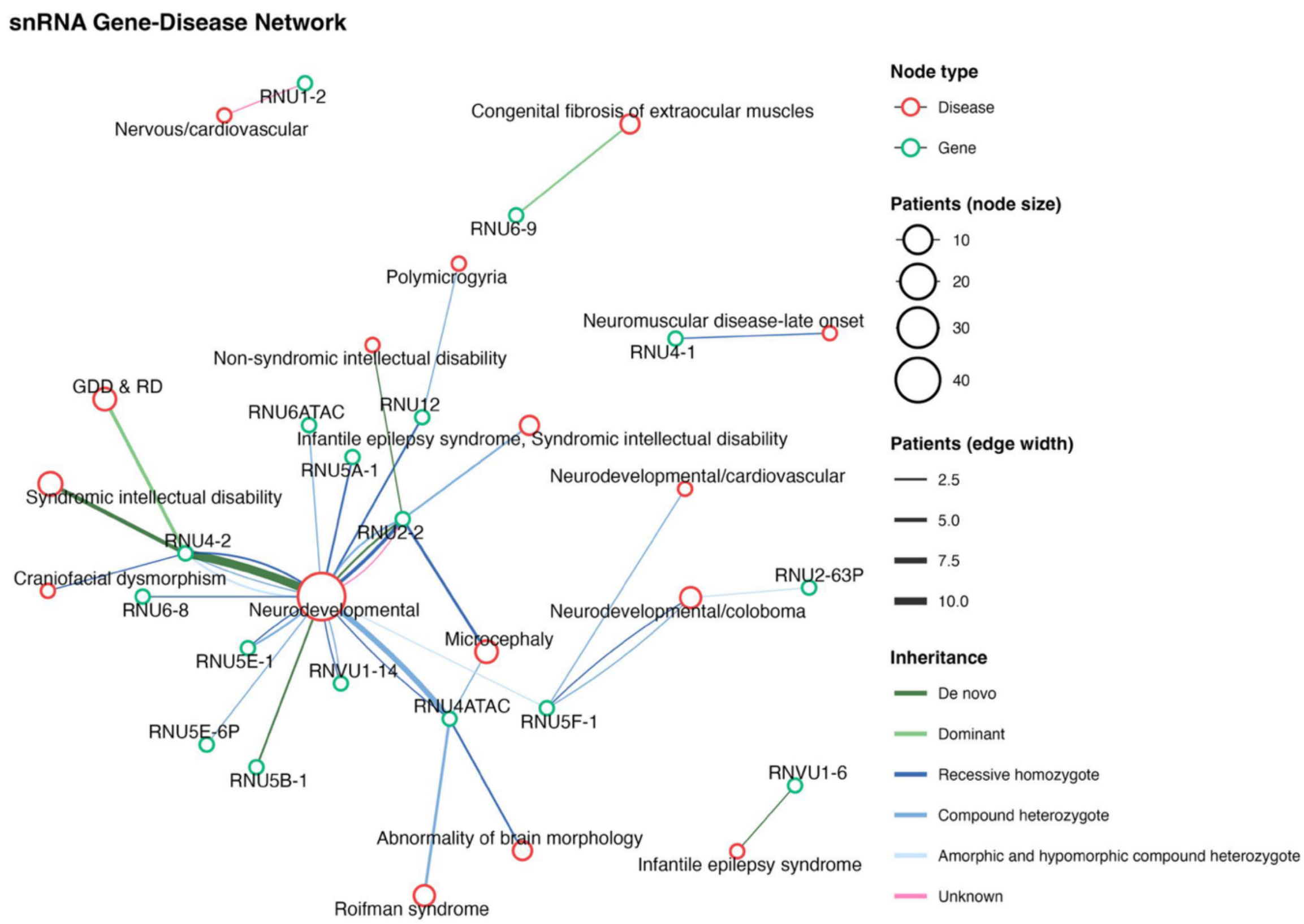

Supplementary Figure 5: Gene-disease network: The graph shows phenotypic heterogeneity and inheritance variability related to RNU genes and pseudogenes. Green and red circles represent genes and diseases, respectively with the size of the latter being proportional to the number of patients having the corresponding phenotype. The width of the lines is proportional to the number of patients in this gene-phenotype inheritance pattern.

medRxiv preprint doi: https://doi.org/10.64898/2026.08.06.26358951; this version posted August 10, 2026. The copyright holder for this preprint (which was not certified by peer review) is the author/funder, who has granted medRxiv a license to display the preprint in perpetuity.
It is made available under a CC-BY 4.0 International license .

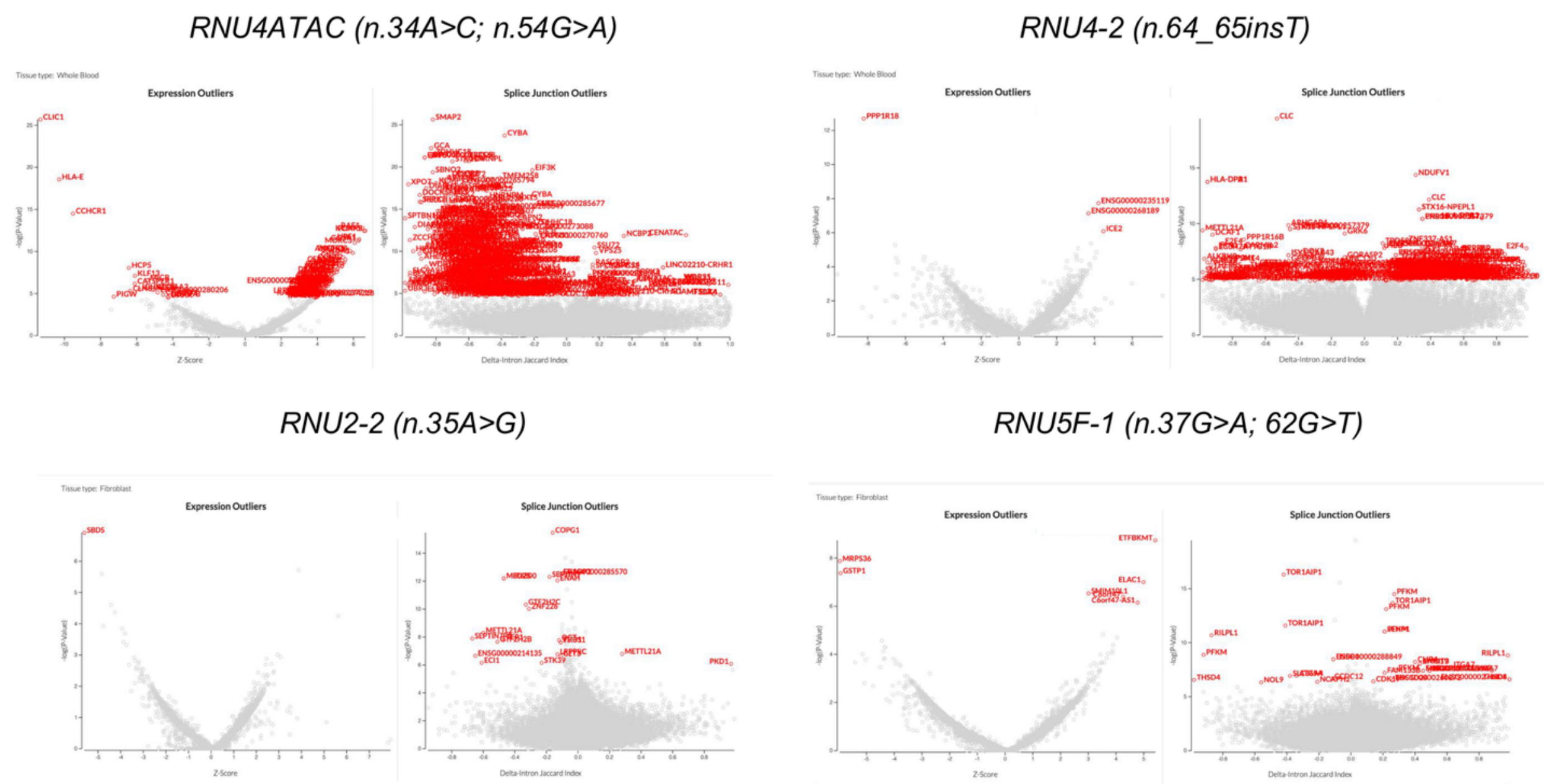

Supplementary Figure 6: RNAseq data analysis: For each panel, volcano plots illustrate OUTRIDER (left) and FRASER2 (right) results. Genes in red correspond to expression and splice junction outliers (SJO), respectively. Diagnostic variants show significant high SJO in *RNU4ATAC* and *RNU4-2*, but not for *RNU2-2*. Results pertaining to a candidate variant in *RNU5F-1* are also shown.
